# 815 Translational Research in Skin of Color Spontaneous Repigmentation of Post-Chemical Burn Leucoderma

**DOI:** 10.1093/jbcr/irac012.363

**Published:** 2022-03-23

**Authors:** Marcelo L Paiva, Helio A Miot, Flavio L Beltrame, Aline S Justo, David T Harrington, Mary A Hunter, Carlos G Wambier

**Affiliations:** The Warren Alpert Medical School of Brown University, Providence, Rhode Island; Department of Dermatology and Radiotherapy, Sao Paulo State University (UNESP), Botucatu, Brazil, Botucatu, Sao Paulo; Department of Pharmaceutical Sciences, State University of Ponta Grossa, Ponta Grossa, Brazil, Ponta Grossa, Parana; Department of Pharmaceutical Sciences, State University of Ponta Grossa, Ponta Grossa, Brazil, Ponta Grossa, Parana; Brown Surgical Associates, Providence, Rhode Island; Department of Surgery, The Warren Alpert Medical School of Brown University, Providence, Rhode Island; Department of Dermatology, The Warren Alpert Medical School of Brown University, Providence, Rhode Island

## Abstract

**Introduction:**

After facial burn injuries to skin of color (SOC) patients, repigmentation plays a critical role in post-burn quality of life, given the acquired contrast between preserved skin and achromic scars, and that daily concealment may be difficult. While leucoderma is a well-known side-effect of deep chemabrasion with phenol, phenol-croton oil, and high concentration trichloroacetic acid, mechanisms of repigmentation are still not fully understood, and the observation of spontaneous repigmentation of a deep chemical injury in SOC has not previously been described in the literature. In our study, we present the dynamics of long-term spontaneous facial repigmentation of a Fitzpatrick V chemical burn patient, with microscopic findings of a deep chemical peel SOC animal model.

**Methods:**

We demonstrate spontaneous repigmentation in a 37-year-old female who initially presented with depigmented areas, erosions and eschars on the face, 7 days after accidental deep chemical burn, due to self-application of 70% glycolic acid to improve acne. This is juxtaposed with a SOC porcine experiment, performed with 35% phenol, 1.6% croton-oil, to evaluate depth-of-injury, neocollagenesis band thickness in 21 days, and skin pigmentary safety. A 6mm punch biopsy taken at 21 days underwent Herovici staining to reveal the thickness of collagen type III neocollagenesis, confirm the presence of melanin in the epidermis, and evaluate the presence of melanophages.

**Results:**

On patient presentation, epidermolysis and areas of deep dermal burns were observed over the forehead and cheeks. At 14 days, multiple leucoderma scars persisted and initial perifollicular repigmentation was noticeable. At 28 days, most of the skin was repigmented; however, many areas had only perifollicular repigmentation and still very severe leucoderma. At 6 months, the skin still had small areas of leucoderma with folicular repigmentation. At 11 months the skin color was fully recovered, with minimal post-inflammatory hyperpigmentation (PIH) over the cheeks.

As for the SOC animal model, at 7 days the eschar was detached, and the skin was achromic. At 21 days, skin pigmentation was completely restored without macroscopic signs of PIH. Dermatopathology evaluation revealed microscopic areas of melanophages, a fully recovered epidermal melanin, and a band of over 500 micra of collagen type III.

Overall, on follicle-dense areas after chemical burn, we observed progressive full repigmentation over the course of 11 months on the patient, and by day 21 in the porcine SOC model.

**Conclusions:**

Spontaneous repigmentation occurred both after accidental deep chemical ablation to the face of a SOC patient, and on the porcine SOC model after deep chemical peel formula application.

